# Do you know this syndrome? Dyspigmentation along the Blaschko lines caused by trisomy 7 mosaicism*

**DOI:** 10.1590/abd1806-4841.20164922

**Published:** 2016

**Authors:** Miguel Pinto de Gouveia, Inês Coutinho, Vera Teixeira, Renata d'Oliveira, Margarida Venâncio, Ana Moreno

**Affiliations:** 1 Coimbra University Hospital Centre (CHUC) – Coimbra, Portugal

**Keywords:** Hyperpigmentation, Hypopigmentation, Mosaicism, Trisomy

## Abstract

Dyspigmentation along the Blaschko lines is strongly suggestive of a mosaic skin disorder. We report a 9-year-old male patient who presented with swirls and streaks of both hypo and hyperpigmentation involving the entire body. Additionally, he had hypertrichosis, musculoskeletal and minor neurodevelopment abnormalities but no intellectual disability. Cultured fibroblast displayed trisomy 7 mosaicism, which can explain this pigmentary phenotype. Widespread dyspigmentation associated with involvement of other organs should prompt systemic examination to detect additional anomalies and genetic evaluation should be considered, even with normal fetal karyotype.

## CASE REPORT

**Figure 1:**
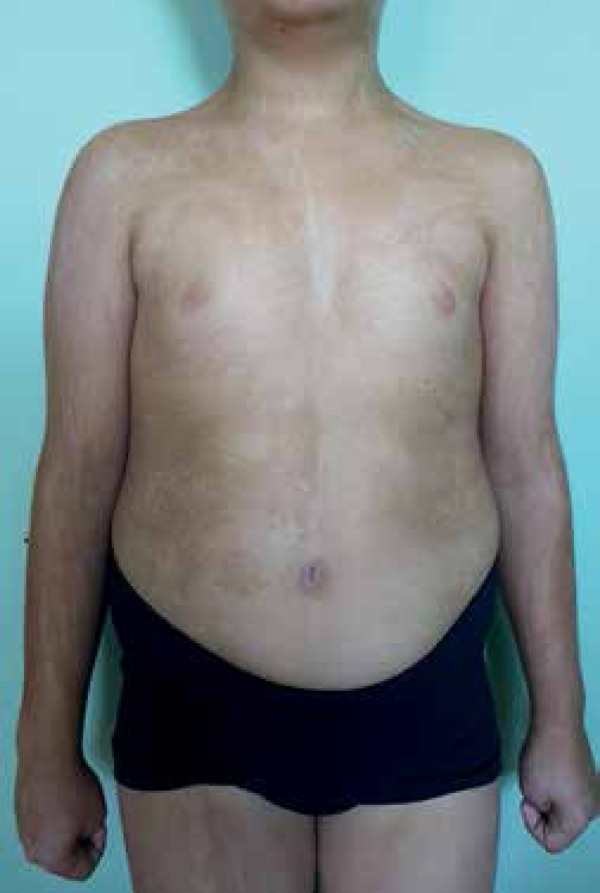
Swirls and streaks of both hypo and hyperpigmentation on the anterior trunk and limbs

**Figure 2:**
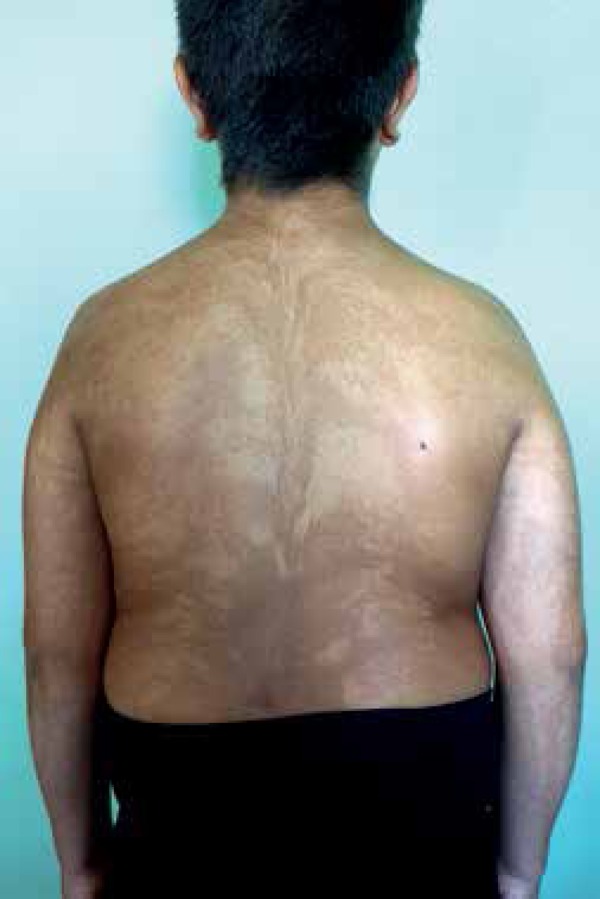
Hypo and hyperpigmentation, sharply demarcated at the midline on the posterior trunk, with associated hypertrichosis

A 9-year-old male with congenital widespread skin dyspigmentation presented with swirls and streaks of hypo and hyperpigmentation, sharply demarcated at the midline on the anterior and posterior trunks (Figures 1 and 2). The patient also displayed hypertrichosis, dorsal scoliosis, mild asymmetrical distortion of the skull, fine motor coordination disturbance and dysorthography.

Medical history revealed amniocentesis performed due to a cystic hygroma in the first trimester of his gestation, which identified a normal karyotype (46,XY). Family history was irrelevant with non-consanguineous parents.

He was referred to the genetics clinic, where a cytogenetic study was conducted on skin fibroblasts. We analyzed 100 metaphases of hyperpigmented skin (A) and 41 metaphases of hypopigmented skin (B). The results revealed trisomy 7 mosaicism in 5% of the metaphases in both fragments [A: 47,XY,+7[5]/46,XY[95] and B: 47,XY,+7[2]/46,XY[39]]. Remaining ancillary exams showed no abnormalities.

The patient is currently being followed by neuropediatricians.

## DISCUSSION

Dyspigmentation along the Blaschko lines is often referred to as hypomelanosis of Ito (HI) – hypopigmentation – or linear and whorled nevoid hypermelanosis (LWNH) – hyperpigmentation. Since it is difficult to determine the baseline skin color, many authors now consider HI and LWNH under the broader concept of pigmentary mosaicism, which can be caused by a wide variety of genetic defects.^1^ Specifically, mosaic autosomal trisomies are responsible for 30% of cases.^2^

Trisomy 7 mosaicism can explain pigmentary phenotype in our patient since chromosome 7 has several pigmentary genes (e.g. GPNMB; EGFR; HGF)^3^ and chromosomal abnormalities can disrupt their expression or function.^3^

Considering the 18 cases of postnatal diagnosis of trisomy 7 reported in the literature, 16 were mosaic and 9 of them presented with pigmentary mosaicism.^4^ Extracutaneous findings in trisomy 7 mosaicism included renal malformation, facial dysmorphism and growth retardation without a typical clinical presentation. In our case, additional findings of hypertrichosis, musculoskeletal and *minor* neurodevelopment abnormalities were evident.

Our patient also displayed a normal fetal karyotype during amniocentesis, reflecting the difficulty of assessing chromosomal mosaicism during pregnancy. Problems include the possibility of a sampling error and the difficulty to distinguish a pseudomosaicism resulting from a culture artefact (in 5% of amniocentesis) of a true chromosomal mosaicism (observed in 0.2% of cases). ^2^

Postnatal karyotyping of at least two tissues may be necessary for a correct diagnosis. Since mosaicism is not always evident in peripheral blood, the procedure is usually performed on fibroblasts from both light and dark skin.^5^

Although there is no specific treatment for widespread dyspigmentation, a meticulous systemic examination should be carried out to detect further abnormalities. Neurology referral and baseline ophthalmology evaluation are advisable to detect subtle development delays or motor defects.^6,7^ Other subspecialty referrals should be individualized, as dictated by clinical findings.

Genetic evaluation, including karyotyping, should be considered in patients with widespread dyspigmentation associated with involvement of other organs,^6^ even with normal fetal karyotype.

## References

[1] Di Lernia V (2007). Linear and whorled hypermelanosis. Pediatr Dermatol.

[2] Hsu LY, Kaffe S, Jenkins EC, Alonso L, Benn PA, David K (1992). Proposed guidelines for diagnosis of chromosome mosaicism in
amniocytes based on data derived from chromosome mosaicism and
pseudomosaicism studies. Prenat Diagn.

[3] Taibjee SM, Bennett DC, Moss C (2004). Abnormal pigmentation in hypomelanosis of Ito and pigmentary
mosaicism the role of pigmentary genes. Br J Dermatol.

[4] Petit F, Holder-Espinasse M, Duban-Bedu B, Bouquillon S, Boute-Benejean O, Bazin A (2012). Trisomy 7 mosaicism prenatally misdiagnosed and maternal
uniparental disomy in a child with pigmentary mosaicism and Russell- Silver
syndrome. Clin Genet.

[5] Hartmann A, Hofmann UB, Hoehn H, Broecker EB, Hamm H (2004). Postnatal confirmation of prenatally diagnosed trisomy 20
mosaicism in a patient with linear and whorled nevoid
hypermelanosis. Pediatr Dermatol.

[6] Treat J (2010). Patterned pigmentation in children. Pediatr Clin North Am.

[7] Chen H, Chen H (2012). Hypomelanosis of Ito. Atlas of Genetic Diagnosis and Counseling.

